# Biological Activity and Chemical Composition of *Detarium microcarpum* Guill. and Perr—A Systematic Review

**DOI:** 10.1155/2022/7219401

**Published:** 2022-10-08

**Authors:** Abdulrahman Mahmoud Dogara

**Affiliations:** Biology Education Department, Faculty of Education, Tishk International University, Erbil, Kurdistan Region, Iraq

## Abstract

Historically, natural products have been the principal source of medications for the treatment of human diseases. Traditional medical practitioners employ *Detarium microcarpum* as a treatment for diabetes, malaria, wounds, inflammation, and even cancer. This study emphasizes the importance of harmonizing *D. microcarpum* research so that results from various sources may be directly compared to reach a scientific conclusion. We searched Google Scholar, Science Direct, Google.com, Wiley, PubMed, Hindawi, and Springer for research papers on *Detarium microcarpum*. This analysis excludes untrustworthy online data, thesis papers, and review publications on *D. microcarpum*. The leaves and stem bark were shown to have high antioxidant, anti-inflammatory, antibacterial, antidiabetic, and anticancer properties. The study also discovered that too much consumption is harmful. Polyphenols and flavonoids were the most commonly reported compounds. However, human safety and efficacy are yet to be fully evaluated, and further well-designed clinical trials are needed to confirm preclinical findings. The leaves and stem bark extracts and isolated compound mechanism of action should be investigated. It is necessary to set a standard dose and ensure its safety.

## 1. Introduction

In recent years, as infectious diseases have spread and harmful organisms have developed resistance to therapy, the quest for new treatments has increased substantially [1]. Humans have always drawn inspiration from plants due to their unique medical characteristics. Traditional African medicine relies heavily on the use of medicinal plants for the treatment of a wide variety of illnesses [2, 3]. Medicinal plants have been used by native peoples for centuries to cure and prevent illness, and they also serve as a source for a wide range of useful pharmaceuticals and other health aids [4]. Due to the rise in infectious diseases and treatment resistance among pathogenic organisms, the search for novel drugs has intensified significantly [5, 6]. Many of the chemical substances found in plants have physiological functions and can be used to treat or prevent disease [7]. Compounds that exhibit biological activity include flavonoids, tannins, saponins, alkaloids, glycosides, and phenolics [8]. Chemical compounds found in plants include a wide variety of physiologically active molecules that can be employed to treat or prevent disease [9]. *Detarium microcarpum* is an African native plant that grows in the wild in several African countries, notably in savannah areas. Because every part of the *D. microcarpum* plant has a therapeutic application, the plant itself is referred to be a miracle plant by the traditional herbalist. Folk medicine relies heavily on its leaves and fruits. The need for new drugs derived from numerous species of medicinal plants is continually growing today [10]. This study aims to bring together the scattered data on the biological impacts of *D. microcarpum* and synthesize it into a cohesive whole, paving the way for a more thorough understanding of the plant and a clearer guidance on how to make the best use of its components. Therefore, traditional applications, taxonomic description, distribution and biological activity, and chemical composition are all documented in the following review.

## 2. Methodology

Inclusion criteria: we searched for articles on *Detarium microcarpum* using various online databases, including Google Scholar, Science Direct, Google.com, Wiley, PubMed, Hindawi, and Springer and only the articles from reliable web sources were included in this research study. These online databases extract useful information from the original scientific research papers that they index. These biological evaluations, along with a great number of others, were used as key terms in our investigation. Antifungal and antibacterial, anti-inflammatory, herbal, and anticancer treatments were among these. While chemical composition phrases such as chemical content, chemical composition, mineral content, mineral analysis, heavy metal composition, heavy metal analysis, HPLC, HPTLC, GCMS, FTIR, and many others were used. Exclusion criteria: this analysis did not include any data obtained from unreliable web sources and also excluded thesis papers and review articles.

## 3. Results and Discussion

### 3.1. Taxonomy, Description, and Distribution

**Figure 1:**
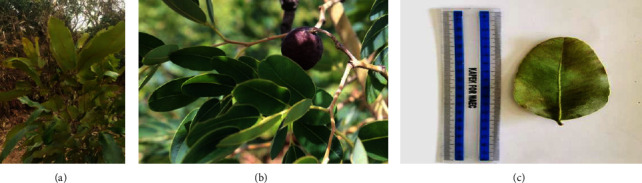
(a) branch (source author), (b) leaves displaying fruit [17], and (c) single leaf (source author).

*Detarium microcarpum* is a part of the broader leguminosae family, *Caesalpiniaceae*. It is a tropical African rainforest and savannah tree [11–13]. *Detarium senegalense* sensu auct is a misapplied name to *D. microcarpum.* It happens normally in the drier parts of Africa [14]. *D. microcarpum* can be found in the arid lands of western and central Africa, from Senegal to Sudan, Nigeria, and even Zaire, where it thrives. It may grow to a height of 36 meters and has a big, dense crown. *D. microcarpum* thrives in dry areas of central and western Africa, reaching heights of up to 15 meters. It can reach a height of 25 meters in locations with a lot of rain. The tree's fractured grey bark and dark green 8–12 cm leaves make it easy to identify. The tree grows in dry Savannah areas of Africa [15, 16], and it can be found in Sudan's Darfur, Blue Nile, and Kordofan states [16]. A grayish-brown color covers the bark, which is rather smooth but is covered in small cracks (Figure 1). From November through March, it produces its berries and nectar which are 4 to 6 cm in diameter, delicious, fibrous, and one seeded [18].

### 3.2. Chemical Composition

**Figure 2:**
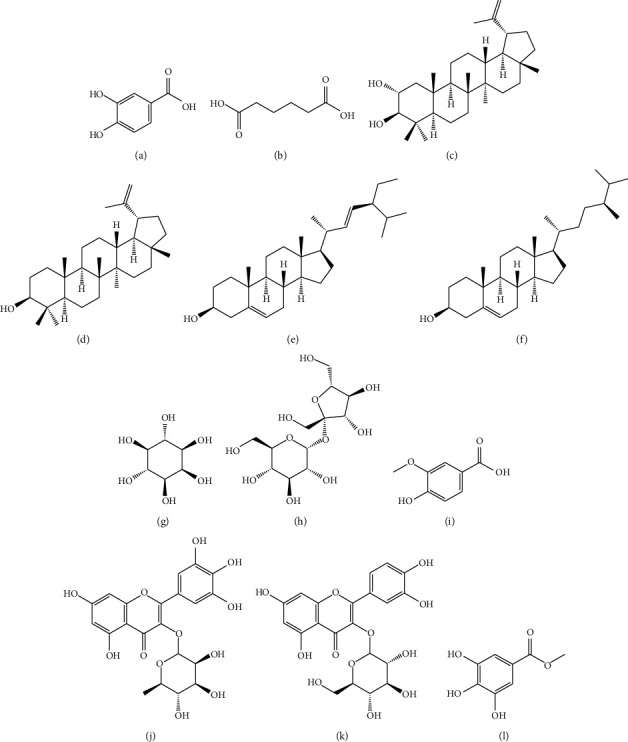
Compounds responsible for the biological activity of the *Detarium microcarpum*: (a) protocatechuic, (b) hexanedioic acid, (c) Lup-20 (29)-ene-2alpha, 3beta-diol, (d) lupeol, (e) stigmasterol, (f) campesterol, (g) myo-inositol, (h) sucrose, (i) vanillic, (j) myricetin 3-O-rhamnoside, (k) quercetin 3-O-glucoside, and (l) methyl gallate.

Many natural product chemists are drawn to the study of physiologically active plant-based natural products on a global scale [19]. Various plants have been evaluated for their biological properties, and in certain instances, active compounds have been found and extracted [20]. The biological activity of medicinal plant parts is due to the amalgamation of diverse substances in the plant, known as secondary metabolites [21]. The majority of these are phenolic compounds, tannins, steroids, and alkaloids, which are produced in various areas of the plant and have various roles [21]. Thousands of compounds have been isolated and identified with the application of contemporary scientific procedures. Many of which have served as chemical leads in the creation of chemotherapy medicines for a variety of ailments [22]. In the chemical investigation, ten components representing more than 99% of the fixed oil were discovered (Table 1 and Figure 2). Three fatty acids were discovered (palmitic, linoleic, and oleic acids) Linoleic acid (44.1%) and oleic acid (30.8%), were the predominant components of the oil, and these unsaturated fatty acids, which have been documented to have antimicrobial properties, may be responsible for the activity seen in the seeds [13]. With three strains of *Salmonella*, the results showed that (1) microcarpamide (2) microcarposide, and (3) rhinocerotinoic acid had a moderate effect [37]. The seed hydrocolloids of *D. microcarpum* were found to contain twenty-two chemicals. They are composed of hydrocarbons 20.32, aromatics 2.14, aldehyde 0.49, phenolic 0.37, fatty acids 67.80, esters 5.09, and alcohols 3.80%, respectively, and these make up the majority of their composition of essential oil [23]. The pure compounds produced by bioassay-guided fractionation were evaluated for growth inhibition and acetylcholinesterase inhibition (Table 1). At 100 *μ*g, compound 2 mildly inhibited *C. cucumerinum* development, while compounds 1 and 5 were moderate inhibitors [24]. At 0.1 *μ*g, compound 2 inhibited acetylcholinesterase as well [24]. In addition to being an acaricid (a drug that kills mites or ticks) for use in orchards, hexanedioic acid, a mono (2-ethylhexyl) ester compound, can also be used as an inert ingredient in insecticides, among other applications [35]. Gallic acid, myricetin 3-O-rhamnoside, quercetin 3,7-O-dirhamnoside, quercetin 3-O-glucoside, and quercetin 3-O-rhamnoside were identified as the five possible radical scavengers found in the precolumn DPPH-HPLC experiment [38]. The nutritional composition of the plant parts was found to be very good for human consumption (Table 1). To sustain normal physiological processes, the human body requires trace amounts of vitamins; therefore, deficiency of vitamins can result in a variety of adverse effects.

### 3.3. Traditional Uses

Ethnomedicinal plants are a major source for novel medication development [46]. Traditional pharmaceutical uses for the plant's roots, leaves, and bark, as well as its high quality firewood, influenced a variety of regional approaches to management [47]. The Detarium microcarpum is frequently used in traditional medicine to treat numerous ailments, including diarrhoea, bronchitis, fever, meningitis, convulsions, malaria, diabetes, bacterial, and fungal infections [11]. One of Africa's most important medicinal plants is Leguminosae *D. microcarpum* [11, 34]. It is believed by the Ibo people, who live in the south-eastern part of Nigeria, that the “Ofo” plant is a “religious” tree that grows in God's own compound and represents sincerity and candor, frequently referred to as sweet dattock or tallow [12]. It is also known by its traditional name, Abu Laila, in western Sudan. In Senegal, it is called dank; in Mali, it is called tamba dala [16, 48]. To prevent infection, wounds can be treated with fresh bark or leaves, and the powder made from boiled bark can be used as a painkiller [49]. An infusion of the bark is reported to be anti-inflammatory, anti-parasitic, and diuretic, while the fruits and leaves are used to treat diarrhea and syphilis [34]. Roots, stems, bark, leaves, and fruits of this plant have been shown to be effective in treating a wide range of ailments, including diarrhea, tuberculosis, and meningitis [16]. People in Mali use the bark to treat measles, while the roots and leaves are used to treat cramps and diarrhoea in humans and cattle, respectively [49]. For skin diseases, the fruit pulp is used in Burkina Faso, and for dizziness, it is used in Niger Republic and Togo [49]. The fruit, leaves, and seeds of this plant are all edible [16]. The bark's infusion is said to have antiparasitic, diuretic, and anti-inflammatory properties, while the fruits and leaves are used to cure diarrhea and syphilis [34]. There are African tribes that use the fruit and leaves as a vegetable and as a seasoning for their food [16, 48]. Diarrhea and syphilis can be treated with bark infusion, while dysentery and syphilis can be treated with fruits and leaves, respectively [12, 50]. *D. microcarpum* is frequently used as a folk remedy to treat ailments due to its medicinal characteristics; it is used to treat diarrhoea, meningitis, TB, and haemorrhoids. The leaves are consumed as a vegetable and are used as an enema for diarrhea, an eye wash for conjunctivitis, and a traditional wash for itch [13]. The bark is used to cure anaemia and to expel the placenta that has been retained. In Senegal, it is used to treat bronchitis, pneumonia, and other respiratory ailments using palm wine maceration [13]. The plants are also used in Nigeria for the treatment of cancer [51]. Women in Sudan use the delicious aroma of heated roots as a perfume, and people in the Chad Republic use it to keep mosquitoes away [49]. Because it lights quickly, especially in the presence of moisture, it is also used for firewood and charcoal [49]. The study substantiated the traditional therapeutic efficacy of *D. microcarpum*, indicating its potential as a source of valuable medicine.

### 3.4. Biological Evaluation

**Figure 3:**
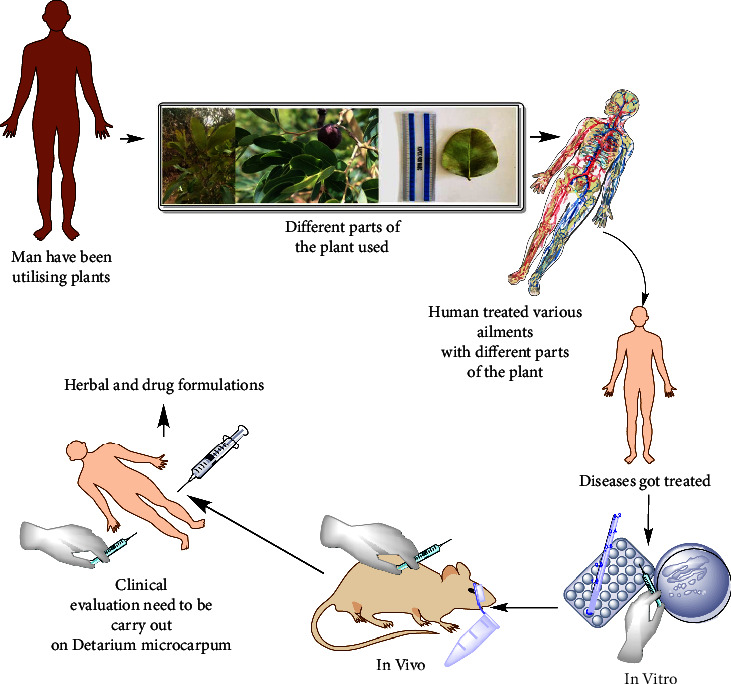
Diagrammatic presentation of how man utilises plants leads to the development of modern drugs and herbal formulations.

Medicinal plants refer to a wide range of plants, some of which have medicinal properties. *Detarium microcarpum* has been utilized as a medicinal herb for decades in various parts of the world to cure and manage various diseases. The intriguing activity of plant parts has prompted scientists from all around the world to examine the biological potential of the plant's numerous sections (Tables 2–10). Despite its *invitro* and *invivo* activities, no clinical study has been conducted yet (Figure 3).

### 3.5. Antioxidants

Oxidative stress is the presence of reactive oxygen species (ROS) and free radicals, both of which are created within normal physiological conditions but become harmful when they are not removed by endogenous systems. ROS and free radicals are also referred to as “reactive oxygen species [97].” Free radicals and reactive oxygen species are produced by the body under normal physiological settings [97]. A chemical molecule is said to be a free radical if it possesses an unpaired electron that is revolving about the molecule's nucleus's periphery [98]. The two sources of free radicals in the body are endogenous sources, such as food metabolism and the aging process, and external sources, such as ionizing radiation, tobacco smoking, organic solvents, air pollution, and pesticides [99]. The search for antioxidants from natural sources has attracted a great deal of interest, and scientists are working diligently to identify molecules that could replace synthetic antioxidants. A wide variety of human activities rely on the secondary metabolites produced by the plant's constituents. It is well-known that these chemical products have a wide range of biological applications [100]. Different methods were used to evaluate the antioxidant potential of *D. microcarpum,* such as DPPH, FRAP, hydrogen peroxide, nitric oxide, ABTS, SRASA, DDA, LPM, and many more methods (Table 1). All parts of the plant have been examined for their antioxidant potential except the root and all are found to exhibit positive results against the free radical (Table 2). In terms of antioxidant activity, all methods showed significant activity (Table 2). The extract had IC_50_ values of 90 *μ*g/mL for hydrogen peroxide and 25 *μ*g/mL for nitric oxide radical scavenging. Hydrogen peroxide was more effectively quenched by the extract (IC_50_ = 90 *μ*g/mL) than gallic acid at *p* > 0.05, although the two compounds exhibited similar quenching abilities [55]. The IC_50_ value of the essential oil extracted from the leaves was 21.99 *μ*L, which is the maximum level of radical scavenging activity [28]. The ethanolic fruit extract had IC_50_ values of 49.87, 69.06, and 49.36 *μ*g/mL in the DPPH, deoxyribose degradation, and lipid peroxidation models, demonstrating its outstanding antioxidant capabilities [53]. It is possible that the chemical contents response to this type of assay is responsible for the observed differences in results. Five compounds have been isolated from *D. microcarpum* leaves: quercetin 3-O-glucoside, gallic acid, myricetin 3-O-rhamnoside, quercetin 3, 7-O-dirhamnoside, and quercetin 3-O-rhamnoside. It was discovered that they all possessed strong radical scavenging activities [38]. Flavonoid and polyphenol content in *D. microcarpum* fruit pulp contributes to its high antioxidant activity [53]. The primary bioactive phytochemicals found in fruits are polyphenols, which have been shown to be successful in preventing some chronic diseases such as diabetes, cancer, and coronary heart disease. Because of this, the consumption of *D. microcarpum* might be beneficial to the health of consumers. Antioxidant properties of *D. microcarpum* were found to be promising, according to the findings. D. microcarpum could be used to produce neuraceuticals. Moreover, the bioactive compounds that were found can be used as good indicators for quality control and should be studied as a potential source of antioxidant-related disorders in the future. Plant extracts or compounds can exert a synergistic effect by chelating metals. When these two types of radicals combine, they form a complex called an antioxidant radical synergist (A : S) in which neither type of radical can catalyse oxidation reactions on its own. The ability of the antioxidant radical to help break down lipid peroxides is inhibited by this chemical association [101]. The number of hydroxyl (OH) groups on the aromatic ring is inversely related to its effectiveness (s). These compounds may also chelate pro-oxidative metals, depending on the configuration of the OH groups [101].

### 3.6. Anti-Inflammatory Activity

Inflammation is a multifaceted phenomenon. It represents the response of the organism to diverse stimuli and is associated with a number of conditions requiring lengthy or recurrent therapy, such as arthritis, asthma, and psoriasis [102]. It is the leading cause of death in the globe [103]. Several medications are used to treat inflammatory diseases, but long-term use can induce gastrointestinal distress, bone marrow depression, and water and salt retention [102, 104]. Research on medicinal plants with anti-inflammatory properties is important. Depending on the experimental model employed, the leaf and stem bark extracts of *D. microcarpum* were revealed to contain unique anti-inflammatory properties (Table 3). The LD_50_ of the extract in the animal model was 471.2 mg/kg body weight when it was injected intraperitoneally, and ≥5000 mg/kg body weight when it was orally administered [62]. When compared with the usual saline-treated group, the methanolic extract reduced the mean diameter of the rat paw in the carrageenan-induced inflammation [62]. At *p* < 0.05, the results showed that methanol leaf extract had a significant dose-dependent anti-inflammatory effect against egg albumin and formalin-induced inflammation [63]. At the 5th hour, the extract suppressed egg albumin by 26.5 and 29.4% at dosages of 200 and 400 mg/kg, respectively. The percent inhibition of formalin-induced edema was 32.5 for all dosages of 200 and 400 mg/kg [63]. The polyphenolic chemicals found in abundance in the studied extract may be responsible for these effects. Gallic acid had anti-inflammatory, antioxidant, antianaphylactic, antitumor, and antiradiation effects in prior research studies [105, 106]. The anti-inflammatory properties of *D. microcarpum* aqueous extracts, which contain polyphenolic compounds such as vanillic acid, gallic acid, and protocatechuic acid, imply that they are promising herbal remedies for pain and inflammation management [107]. Increased blood flow, increased cellular metabolism, vasodilatation, release of soluble mediators, extravasation of fluids, and cellular influx are all common symptoms of inflammation, and these mechanisms are the same and are independent of the triggering cause [108]. Arachidonic acid and inflammatory mediators such as cytokines, serotonin, histamine, prostaglandins, and leukotrienes are released from cells in response to the inflammatory agent, which also increases vascular permeability and facilitates leukocyte migration to the site of inflammation [108].

### 3.7. Antibacterial Activity

Since the beginning of human history, infections have been treated with medicinal plant extracts. Antimicrobial drugs are crucial to lowering the global burden of infectious diseases [109]. However, when resistant organisms evolve and spread, antibiotics lose their potency [110]. This type of bacterial resistance to antimicrobial agents poses a very serious threat to public health, and the frequency of resistance is increasing worldwide for all types of antibiotics, including the major last-resort medications [110]. Consequently, other antimicrobial antibiotics are urgently required, and this circumstance has prompted evaluation of the therapeutic use of ancient remedies. The agar disc diffusion methods are mostly used to determine if bacteria are responsive to antimicrobial drugs as a qualitative test. The MIC, MBC, broth dilution, and many other methods, refers to the amount of substance required to prevent bacterial growth or the amount bacterial concentration needs to be used. Several studies explored the antibacterial properties of various parts of *D. microcarpum* using different methods (Table 4). The stem bark ethanolic extracts demonstrated antibacterial activity against the investigated species at dosages of 100, 50, 25, and 12 mg/mL, with *S. aureus* having the highest zone of inhibition at 21 mm at 100 mg/mL [44]. At 13 mm, the ethanolic bark extract showed the most efficacy against *Listeria monocytogenes* [33]. The seeds' petroleum ether extract was found to be effective against all eight strains tested, with the largest zone of inhibition against *S. aureus* measuring 8.8 mm [26]. *Detarium microcarpum* dichloromethane extracted was the most efficient at 75 percent in preventing *Pythium aphanidermatum* growth [68]. According to the documented study (Table 4), bacterial resistance to *D. microcarpum* parts has never been reported, making it a very promising antibacterial agent. Isolated from the fruits were compounds with significant antimicrobial activity against the strains used in the study, including microcarposide. Lupeol, betulinic acid, -sitosterol glucoside, methyl gallate, [45] humulene-1,2-epoxide, caryophyllene oxide, neryl acetone-caryophyllene, salvia-4 (14)-ene1-one, and linalool are some of the main components of the essential oil of *D. microcarpum* leaves, which have antimicrobial properties [28]. This does not rule out the potential that other components, either alone or in combination with important molecules, do not contribute to these biological features. It was discovered that the presence of secondary metabolites, which had the power to stop the growth of particular bacterial strains, was responsible for the antibacterial action of therapeutic plant extracts and essential oils [111]. By disrupting the lipid part of the microorganism plasma membrane, a plant extract's antimicrobial activity causes membrane permeability to change and intracellular materials to seep out [111]. The capacity of the discovered compounds to permeabilize and depolarize the cytoplasmic membrane was linked to their antibacterial action against the tested strains. Compounds also interfered with signalling pathways, decreased membrane integrity, inhibited the quorum sensing system, and made the bacterial cell membrane more permeable [112].

### 3.8. Antifungal Activity

Recently, the use of various natural plant compounds to suppress the pathogenic organisms has evolved [8, 110]. Plant products are important sources of therapeutic medications for infectious diseases, and they are reported to have little or no adverse effects when compared to synthetic drugs [113]. Fungal infections cause a wide range of disorders, including aspergillosis, candidiasis, dermatophytosis, and mucormycosis [113]. Plant extracts are frequently employed as a source of medicinal agents, mostly for antifungal purposes. This is due to an increase in the demand for natural products to replace synthetic chemical substances. Antibiotic overuse frequently led to the emergence of resistant strains. Due to this drug resistance, the quest for novel antibiotics is ongoing. In this regard, plants remain as an abundant supply of therapeutic medications. The methanolic stem bark inhibited *Microsporum canis* the most (10.70 mm) [70]. The contractile amplitude of jejunal tissue was lowered by the aqueous extract alone in a dose-dependent manner. Furthermore, an isolated jejunal segment subjected to 0.2 mL of 10 g/mL acetylcholine had its contractile amplitude reduced by the aqueous extract [72]. Isolated molecules of clerodane diterpenes from the pulp exhibited antifungal action and inhibition of the Alzheimer's disease-linked enzyme acetylcholinesterase [53]. The extract dramatically increased the growth of all fungal strains examined (Table 5). The plants exhibited a wide spectrum of activities. The presence of secondary metabolites, which inhibited the growth of microbial strains, was linked to the antimicrobial activity of medicinal plant extracts. The activity was caused by a change in the lipid fraction of the microorganism plasma membrane, which altered membrane permeability and allowed intracellular materials to leak [111]. The reported study demonstrates that the crude extracts are antifungal against all strains tested. This research could lead to the identification of new antifungal drugs and new antibiotic compounds.

### 3.9. Antiparasitic Activity

More than 90 countries with a combined population of 2.4 billion are plagued by malaria [46]. Children under 5 and pregnant women account for the vast majority of the 500 million clinical cases and 1.5–2.7 million annual deaths caused by this disease [114]. African trypanosomiasis, often known as “sleeping sickness” in humans and “nagana” in animals, is a significant infection that affects both humans and livestock in Africa [115]. The methanolic stem bark extract demonstrated a substantial curative and prophylactic effect at 200, 400, and 800 mg/kg tested doses, and the survival span of mice was considerably (*p* < 0.001) extended in the treated groups. At *p* > 0.05, the extract had no effect on the biochemical and haematological assays (Table 6). The oral LD_50_ of the methanolic leaf extract was found to be larger than 5000 mg/kg. The extract demonstrated significant curative, suppressive, and preventive effects at all doses tested at *p* > 0.001. Furthermore, compared to the negative control group, the extract enhanced the survival time of the treated mice by up to 19 days [76]. When compared to the negative control, both the aqueous and methanolic stem bark extracts restricted larval movement in *C. elegans* Bristol and *C. elegans* DA1316 [77]. However, all studies reviewed revealed that the plant demonstrated good activity against the tested parasites. It is interesting to note that the different antiparasitic effects of these extracts were related to both their chemical composition and the nature of the promastigote species, and that the different antiparasitic activity of these extracts against the different promastigote species is related to their different chemical compositions [112]. The mechanism of action of these extracts can be inferred from their ability to break the cell membrane and induce cell death in certain cellular targets. In this instance, the contact with the mitochondrial membrane can be suggested as an additional targeted method that induces parasite apoptosis.

### 3.10. Antidiabetic Activity

**Figure 4:**
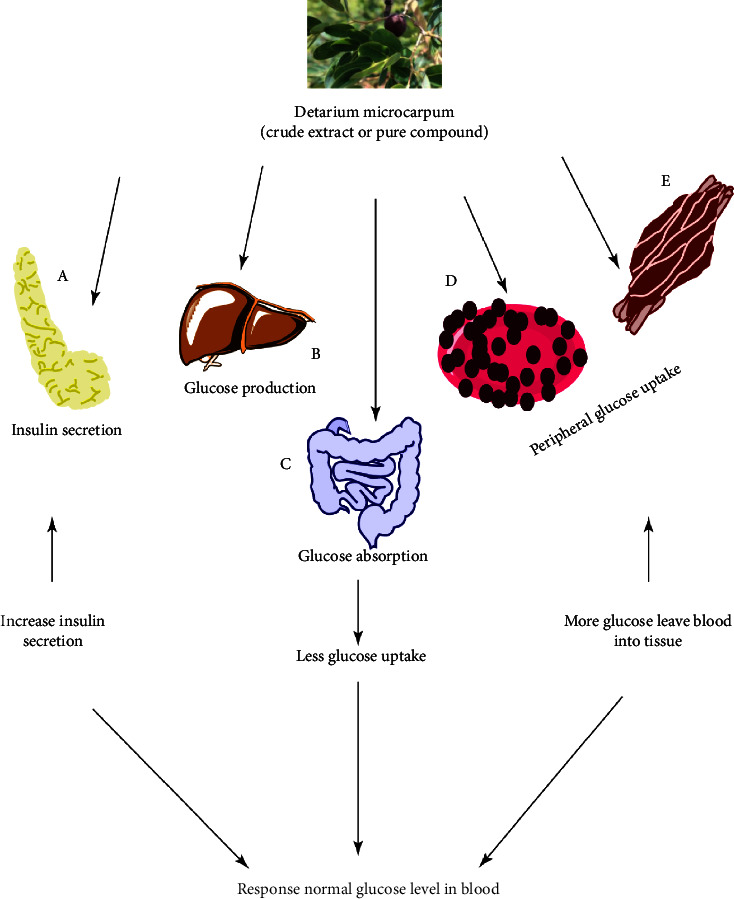
Hypoglycaemic mechanism of *Detarium microcarpum* and effect on target tissues. A = pancreas; B = liver; C = intestine; D = adipose tissue; and E = muscle tissue.

Diabetes mellitus is a prominent and widespread disease that affects populations of underdeveloped, developed, and developing nations [110]. This disease is predicted to afflict 25 percent of the world's population [116]. Diabetes is characterized by improper glucose metabolism, which is connected with low blood insulin levels [116]. The search for novel medications continues as demand grows daily. At *p* < 0.05, the root extract significantly reduced blood sugar levels in alloxan diabetic rats [15]. Seed extract was found to lower blood sugar and cholesterol levels significantly. The extract had no effect on haematological or blood chemical signs, indicating that it was completely safe [84]. The alpha-amylase and glucosidase levels in the methanolic seed extract were reduced to 69.3 and 31.1 mg/mL, respectively [60]. In humans, the extract from the seeds decreases the postprandial blood glucose and insulin levels (Table 7). At *p* < 0.05, the reduction in incremental blood glucose and postprandial glucose levels was statistically significant [87]. The area-under-curve of glucose was 62% [87]. As a result, *Detarium microcarpum* has been studied *in vitro* and *in vivo* as a possible source of antidiabetic medicine. The trunk bark contained myo-inositol (4), L-quino-1,5-lactone (1), D-pinitol (3), D-(-)-bornesitol (2), sucrose, D-glucose, and D-fructose [34]. Actually, D-pinitol and its derivatives are well known for their helpful effects in cases of insulin resistance. The hypoglycemic effects of *D. microcarpum* crude extract and compounds are likely achieved by preventing glucose absorption in the small intestine, increasing insulin secretion in the pancreas, thereby preventing glucose production in the liver, or promoting glucose uptake in peripheral tissues via glucose transporters (Figure 4).

### 3.11. *Detarium microcarpum as* an Insecticide

The ongoing use of liquid and gaseous insecticides is crucial for keeping insect populations in stored products under control [117]. Although effective, their frequent usage for several decades has disrupted the biological control mechanisms of natural enemies and led to outbreaks of insect pests, widespread development of resistance, unwanted effects on nontarget organisms, and environmental and human health issues [117]. The abundance of bioactive compounds in plants suggests they could be a useful alternative to conventional pesticides (Table 8). It contains the active ingredients such as 3,13E-clerodien-15-oic acid, 4 (18)-clerodien-15-oic acid, 18-oxo-3,13E-clerodien-15-oic acid, and 2-oxo-3,13E-clerodien-15-oic acid. Except for the last, these chemicals have not previously been associated with D*. microcarpum*. At a concentration of just 1%, all four substances demonstrated significant antifeedant efficacy [41]. More research is needed to confirm whether or not the plant extracts are effective at controlling pesticides.

### 3.12. Anticancer Activity

**Figure 5:**
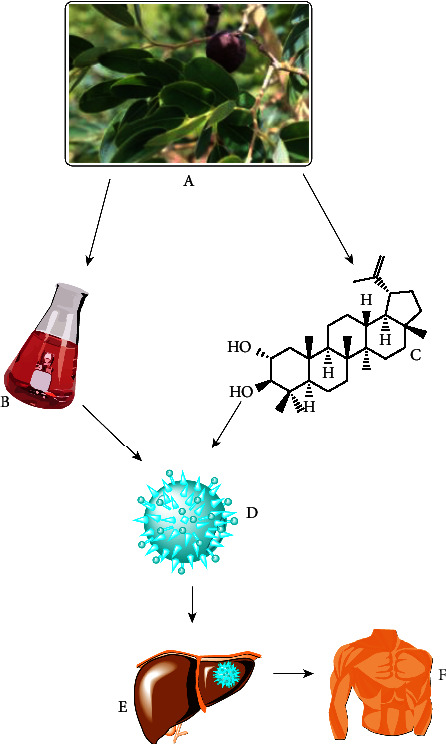
Schematic presentation of *Detarium microcarpum* to target cancer cells. A = *Detarium microcarpum*; B = crude extract; C = pure compound; D = cancer cell; E = organ with cancer cell; and F = treated human.

Cancer is a disease in which cells divide improperly and uncontrollably. In 2012, there were around 14 million new cancer cases and 8.2 million cancer-related deaths globally [118]. Cancer is the world's second biggest cause of death, after only cardiovascular disease, and it is a serious public health concern [118]. Cancer incidence and mortality are increasing all over the world [116]. Further biological study of medicinal plants with anticancer properties will help treat and manage the condition. The IC_50_ values for the three plants' methanol and aqueous extracts inhibiting MCF7 cell growth ranged from 78 to >500 *μ*g/mL. Stem bark extracts had the strongest antioxidant and antiproliferative properties (Table 9). As a result, anti-breast cancer compounds may be found in the stem bark of these plants [59]. dAgNps inhibited HeLa cell proliferation with IC_50_ values of 31.5 *μ*g/mL [79]. The chloroform and ethyl acetate extracts were the most effective at inhibiting osteosarcoma cells. Ethyl acetate extract killed all osteosarcoma cells at all dosages, but chloroform extract killed all cells at concentrations of 250 and 500 *μ*g/mL [96]. Apoptosis is inhibited by plant extracts and chemicals (Figure 5), which also improve survival signalling pathways and disrupt proapoptotic intermediates. Bioactive substances have the potential to affect the angiogenesis pathway, which is the growth of blood vessels in the tumor and is a significant step towards metastasis.

### 3.13. Toxicity Evaluation

Medicinal plants are used throughout the world, particularly in developing countries. This is due to the fact that they are inexpensive and readily available locally. Consumers all throughout the world believe that herbal medicines are always safe since they are natural. Evidence, however, suggests different. If not correctly selected and prepared, they can be highly poisonous. As a result, determining the safety of plant extracts is critical. Many investigations have shown that medicinal plants contain a diverse range of chemicals with biologically beneficial effects. Methanolic leaf extract at a level of 5000 mg/kg has been found to be safe in toxicological tests [63]. At *p* > 0.05, seeds in the diet showed no effect on haematological or biochemical indices. To summarize, tallow seed meal can be used up to 20% in broiler chicken feed without influencing organ weight, haematological index, or biochemical indices (Table 10). Long-term usage of the methanolic stem bark extract in the treatment of illness conditions has been linked to some negative effects on various critical organs [12]. The findings suggest that *D. microcarpum* fruit may have a detrimental effect on rats and may include antinutrients that damage digestion and absorption, resulting in the observed growth retardation in rats [92]. The LC_50_ of the methanolic extract of the stem bark for brine shrimp larvae was found to be 158.49 g/mL. *Detarium microcarpum* is toxic and thus not safe, according to the findings, especially when given in large dosages without effective monitoring and treatment [95]. The methanolic stem bark extract killed mice at doses of 2900 mg/kg and 1600 mg/kg body weight, with an LD_50_ of 3,807.89 mg/kg. Experiments with extracts of these plants have given a favourable dose recommendation for an ongoing antimalarial study [43]. According to the conclusions of this study, excessive usage of all sections of *D. microcarpum* extract may have toxicological implications; hence, only small doses should be utilized. Individual compounds should be examined for their toxicity levels, which is the foundation for any drug development or herbal formulation.

## 4. Conclusion and Future Research

*Detarium microcarpum* has been utilized as an ethno medicine for the treatment of numerous disorders in West Africa and other parts of the world, most notably in Nigeria, Senegal, Sudan, and Mali. Traditional healers treated ailments such as diabetes, malaria, weakness, skin infections, urinary problems, and diarrhea with D. microcarpum leaves, bark, roots, and fruits. Preclinical research has been done on antioxidants, antibacterial, antifungal, antiviral, and treatment for a number of other diseases. This analysis also shows that D. microcarpum has a lot of chemical compounds (Lup-20(29)-ene-2alpha,3beta-diol, Microcarpin, Linoleic Acid, quercetin 3,7-O-dirhamnoside, myricetin 3-O-rhamnoside, and many others) that could be used to make new drugs in pharmaceutical companies and herbal formulations. The study will pave the way for additional research into the isolation of active secondary metabolites and their mechanisms of action against the diseases in question. In-depth studies are required to fully understand the therapeutic applications of the isolated compounds, including their toxicological profiles, mechanisms of action, and their biological activity clinically.

## Figures and Tables

**Table 1:** Chemical composition of *Detarium microcarpum*.

S/N	Compound	Composition	Part of the plant	Solvent	Reference
1	Cyclohexanone	1.4	Seeds	Petroleum ether	[13]
	cis-Rose oxide	0.5			
	Citronellol	8.7			
	*β*-Myrcene	1.4			
	Camphor	0.2			
	Isoledene	1.4			
	Oleic acid	30.8			
	Palmitic acid	0.2			
	E-citral	1.3			
	Linoleic acid	44.1			

2	Nonane	6.52	Seeds	Chloroform	[23]
	3,5-Di-tert-butylphenol	0.37			
	4-Methyldecane	1.27			
	Isopropylbenzene	1.55			
	Decane	7.66			
	Undecane	1.44			
	Hexadecane	0.60			
	3,3-Dimethyl-1-(2-carboxyphenyl) triazene	0.59			
	2,4-Decadienal	0.49			
	2,6,11-Trimethyldodecane	1.11			
	Hexadecanoic acid	9.77			
	Tetradecanoic acid	2.48			
	Hexadecane	1.06			
	9-Octadecenoic acid	39.24			
	Sulfurous acid,2-ethylhexyl isohexyl ester	1.49			
	9,12,15-Octadecatrienoic acid	6.21			
	Pentafluoropropionic acid and hexadecyl ester	1.33			
	Octadecanoic acid	8.12			
	2,6,10,14,18,22-Tetracosahexaene (squalene)	0.66			
	2-Methyl-3,13-octadecadienol	3.80			
	Docosanoic acid	1.98			
	Sulfurous acid, octadecyl-2-propyl ester	2.27			

3	5*α*, 8-*α* (2-Oxokolavenic acid (2)		Fruits pulp	Dichloromethane	[24]
	3,4-Dihydroxy clerodan-13E-en 15-oic acid (4)				
	2-Oxokolavenic acid (3)				
	3,4-Epoxy clerodan-13E-en-15-oic acid (1),				
	3,4-Dihydro clerodan-13z-en-15-oic acid (5)				

4	Calcium	17.97	Fruits pulp (mg/100 g)		[25]
	Chromium	0.44			
	Cobalt	0.06			
	Iron	78.71			
	Potassium	908.1			
	Copper	0.59			
	Iodine 2	2.77			
	Manganese	5.95			
	Molybdenum	6.39			
	Sulphur	37.24			
	Magnesium	113.50			
	Phosphorous	204.5			
	Zinc	31.7			
	Sodium	15.09			
	Cadmium	0.03			
	Nickel	1.57			
	Titanium	0.36			
	Lanthanum	0.09			
	Barium	0.58			
	Strontium	0.25			
	Lead	0.002			
	Arsenic	0.44			
	Vitamin B_2_	4.20	Mg/100 g		
	Vitamin C	55.10	Mg/100 g		
	Folic acid	0.17	Mg/100 g		
	Vitamin E	12.44	Mg/100 g		
	Ash	4.47			
	Crude fibre	12.19	%		
	Crude fat	2.23	%		
	Total carbohydrate	65.8	%		
	Crude protein	4.68	%		
	Cyanide	0.07	Mg/100 g		
	Saponins	2.73	Mg/100 g		
	Phytates	0.41	Mg/100 g		
	Tannins	0.17	Mg/100 g		
	Oxalate	1.06	Mg/100 g		
	Tannins	Present	Seeds		[26]
	Flavonoids				
	Alkaloids				
	Fatty acids				
	Saponins				
	Phenol				
	Steroid				
	Protein	29.4	Fruits %		[16]
	Fat	1.59			
	Moisture	5.74			
	Ash	2.6			
	Fiber	19.05			
	Carbohydrate	41.6			
	Potassium	1463.25	Mg/100 g		[16]
	Magnesium	12.20			
	Sodium	420.50			
	Calcium	136.12			
	Iron	2.73			
	Zinc	0.41			
	Phosphorous	1.05			
	Manganese	2.65			
	Copper	0.35			
	Nickel	0.001			
	Cobalt	2.71			
	Cadmium	0.002			
	Aluminium	1.12			
	Alkaloids	0.37	Seeds %		[18]
	Tannins	0.47			
	Saponins	1.85			
	Flavonoids	2.28			
	Phenols	0.35			
	Alkaloids	0.72	Stem bark %		
	Saponins	4.60			
	Flavonoids	5.68			
	Tannins	0.79			
	Phenols	0.67			
	Magnesium	0.32	Seeds %		
	Potassium	0.50			
	Calcium	1.44			
	Phosphorus	1.0			
	Sodium	0.53			
	Iron	7.11			
	Zinc	5.40			
	Manganese	0.45			
	Potassium	0.50	Stem bark %		
	Magnesium	0.40			
	Sodium	0.40			
	Calcium	1.44			
	Phosphorous	0.54			
	Manganese	0.70			
	Iron	6.97			
	Zinc	6.15			
	Riboflavin	0.62	Seeds mg/100 g		
	Ascorbic acid	83.6			
	Thiamin	0.14			
	Niacin	2.60			
	Ascorbic acid	24.2	Stem bark mg/100 g		
	Riboflavin	0.67			
	Niacin	8.11			
	Thiamin	0.27			
	Crude protein	20.5	Seeds %		
	Fats/oil	55.6			
	Crude fiber	10.5			
	Food energy g/calories	616			
	Ash	840			
	Carbohydrate	840			
	Food energy g/calories	324.6	Stem bark %		
	Fats/oil	55.6			
	Crude protein	9.6			
	Ash	5			
	Crude fiber	17.8			
	Carbohydrate	63.54			
	Protein	27.1	Seed %		[27]
	3-octanone	0.229	Leaves		[28]
	Linalol	2.098			
	Myrcene	0.064			
	Borneol	0.161			
	*α*-Terpineol	0.351			
	*α*-Cubebene	0.357			
	Safranal	0.407			
	Geraniol	0.227			
	1,2-dihydro-trimethyl-naphtalene	0.339			
	*α*-Copaene	1.238			
	*α*-Cubebene	0.357			
	*β*-bourbonene	0.758			
	*β*-Copaene	0.883			
	*β*-bourbonene	0.477			
	*β*-Caryophyllene	11.894			
	Neryl acetone	2.452			
	*α*-trans-bergamotene	0.368			
	Neryl acetone	2.452			
	*γ*-muurolene	1.589			
	*β*-selinene	1.108			
	*α*-humulene	0.605			
	Germacrene-D	0.597			
	*γ*-cadinene	1.13			
	*α*-muurolene	1.027			
	*α*-calacorene	0.73			
	Intermedeol	1.208			
	Caryophyllene oxide	28.186			
	Salvia-4 (14)-ene-1-one	2.243			
	Iso-spathulenol	0.989			
	*δ*-cadinene	1.254			
	Spathulenol	1.886			
	Humulene-1,2-epoxide	4.05			
	1-Naphthaleneacetic-5-carboxy-l,2,3,4,4a,7,8,8a-octahydro-1,2,4a-trimethyl acid		Bark	Chloroform	[29]
	l-Naphthaleneacetic-7-oxo-l,2,3,4,4a,7,8,8a-octahydro 1,2,4a, stetramethyl acid				
	2-Oxo-kolavenic acid				
	Moisture (%)	4.98	Seed		[30]
	Protein (%)	14.22			[30]
	Ash	2.54			
	Crude fat %	8.43			
	Sodium	28.29	Mg/kg		
	Calcium	2700			
	Potassium	23900			
	Magnesium	700			
	Iron	6			
	Zinc	0.4			
	Manganese	1.7			
	Copper	0.2			
	Moisture content (%)	4.69			[31]
	Organic matter content (%)	97.23			[31]
	Protein content (%)	35.96			
	Ash content (%)	2.77			
	Lysine	2.14	g/100 g		
	Leucine	2.35			
	Valine	2.13			
	Isoleucine	2.35			
	Phenylalamine	2.58			
	Methionine	0.74			
	Threonine	2.17			
	Arginine	5.66			
	Cystine	1.07			
	Glutamic acid	9.78			
	Aspartic acid	4.79			
	*Glycine*	2.41			
	Histidine	1.13			
	Tyrosine	1.06			
	Proline	2.09			
	Serine	3.12			
	Alanine	2.10			
	Potassium	105			[32]
	Sodium	002			
	Calcium	0.23			
	Iron	003			
	Phosphorus	001			
	Sulphur	001			
	Iodine	5.420			
	Manganese	2.78			
	Chromium	0.96			
	Molybdenum	0.67			
	Selenium	0.035			
	Zinc	0.635			
	Lead	0.074			
	Arsenic	0.051			
	Copper	0.127			
	Tin	0.362			
	Nickel	0.18			
	Vanadium	0.753			
	Bromine	0.023			
	Cobalt	0.817			
	Strontium	0.083			
	Rubidium	0.914			
	Zirconium	0.025			
	Thallium	1.960			
	Niobium	0.017			
	Yttrium	0.063			
	Sitosterol 3-O-(6′-O-palmitoyl-2,3,4-O-triacetyl-beta-D-glycopyranoside) 1,		Bark	Ethanol	[33]
	Lupeol				
	Lup-20 (29)-ene-2alpha,3beta-diol				
	Stigmasterol				
	Campesterol				
	L-Quino-1,5-lactone		Bark	Ethanol	[34]
	D-(−)-bornesitol				
	Myo-inositol				
	D-pinitol				
	Sucrose				
	D-glucose				
	D-Fructose benzoates				
	Hexanedioic acid		Root		[35]
	Mono (2-ethylhexyl) ester				
	1,2-Benzenedicarboxylic acid		Bark		
	Mono (2-ethylhexyl) ester				
	D-Mannose				[36]
	D-Glucose				
	Labdane diterpenoid		Root	Ethanol/water (7 : 3)	[37]
	Microcarpin (1)				
	Microcarpamide (**2**)				
	5-(Carboxymethyl)-5,6,				
	8a-Trimethyl-3,4,4a,5,6,7,8,8a				
	Octahydronaphthalene-1-carboxylic acid (**3**),				
	Microcarposide (**4**),				
	Rhinocerotinoic acid (**5**),				
	1,7-Dihydroxy-6-methylxanthone (**6**),				
	Ursolic acid (7),				
	3*β*,23-Dihydroxylup-20 (29)-en-28-oic acid (8),				
	Alphitolic acid (9),				
	Stigmasterol glucoside (10)				
	Gallic acid		Leaves	Methanol	[38]
	Quercetin 3,7-O-dirhamnoside				
	Myricetin 3-O-rhamnoside				
	Quercetin 3-O-glucoside				
	Quercetin 3-O-rhamnoside				
	Iron	218.9	Stem bark mg/kg		[39]
	Manganese	139			
	Zinc	48.9			
	Methyl gallate		Stem bark	70% Methanol.	[40]
	Catechin gallate				
	3,13E-Clerodien-15-oic acid, 4 (18)		Leaves	Methanol	[41]
	13E-Clerodien-15-oic acid, 18-oxo-3				
	13E-Clerodien-15-oic acid and 2-oxo-3,				
	13E-Clerodien-15-oic acid				
	Alkaloids		Stem bark	Methanol	[42]
	Flavonoids				
	Glycosides				
	Triterpene				
	Saponins				
	Tannins				
	Indole alkaloid		Stem bark	Methanol	[43]
	Quinoline alkaloids				
	Steroids				
	Steroids				
	Tropane alkaloids				
	Flavonoids				
	Tannins				
	Tannins,		Stembark	Ethanol	[44]
	Saponin,				
	Steroids,				
	Flavonoids,				
	Glycosides,				
	Phenols				
	Terpenoids.				
	Microcarposide (**1**)		Fruits		[45]
	Lupeol (**2**)				
	Betulinic acid (**3**)				
	*β*-Sitosterol glucoside (**4**)				
	Methyl gallate (**5**)				
	Luteolin (**6**)				
	Epicatechin (**7**),				

Notes: S/N = Serial Number.

**Table 2:** Antioxidant activity of *D. microcarpum*.

S/N	Method	Solvents	Part of the plant	Major findings	Reference
1	The standard comet assay	Ethanol	Fruit pulp	DNA integrity was unaffected by fruit pulp extract at concentrations of up to 500 *μ*g/mL compared to vehicle. Extract pretreatment reduced the genotoxicity of hydrogen peroxide and methyl methane sulfonate on human lymphocytes.	[52]
2	*DPPH, FRAP, ABTS, SRASA, DDA, LPM*	Ethanol	Fruits	DPPH, deoxyribose degradation, and lipid peroxidation models showed that ethanolic fruit extract had remarkable antioxidant properties with IC_50_ values of 49.87, 69.06, and 49.36 *μ*g/mL, respectively.	[53]
3	Lipid peroxidation activity	Methanol	Leaves	FeSO_4_/SNP were found to have an inhibitory effect on lipid peroxidation in the brain, liver, and colon when the extract was used as a pro-oxidant. Malondialdehyde content was significantly increased in the colon.	[54]
4	DPPH, FRAP		Essential oil (leaves)	The essential oil extracted from the leaves showed the highest radical scavenging activity with IC_50_ value of 21.99 *μ*L.	[28]
5	Hydrogen peroxide and nitric oxide radical-scavenging assays	Ethanol	Fruit pulp	IC_50_ values of 90 *μ*g/mL for hydrogen peroxide and 25 for nitric oxide radical scavenging were obtained for the extract. It is interesting that the extract had a stronger scavenging activity for hydrogen peroxide than gallic acid (IC_50_ = 90 *μ*g/mL), although the two compounds had similar activity for quenching of nitric oxide radicals at *p* > 0.05.	[55]
6	*In Vivo*	Ethyl-acetate and n-butanol	Stem bark	Significantly improved liver damage and decreased ALT, AST, TBIL, and CBIL values. SML or SMS extracts considerably enhanced glutathione levels in the cell, catalase and superoxide dismutase activities, and greatly lowered reactive thiobarbituric acid components.	[56]
7	*In Vitro*		Fruits pulp	Inhibiting the activity of the enzyme acetylcholinesterase responsible for Alzheimer's disease.	[24]
8	*DPPH*	70% methanol	Leaf	At all doses used, extracts considerably outperformed the reference compounds in terms of radical scavenging activity (IC_50_) at 15 *μ*g/mL.	[57]
9	*DPPH*	Ethanol	Leaves	The ethanolic extract's ability to neutralize free radicals with an IC_50_ value of 4.84 mg/mL.	[58]
10	*DPPH/ABTS*	Methanol and aqueous	Stem bark	The DPPH and ABTS tests had a high degree of correlation (*R*^2^ = 0.7), indicating that they were both trustworthy and significant. As a result, the results indicated that each of the six extracts has significant and dose-dependent antioxidant capacity.	[59]
11	*DPPH, FRAP, ABTS*	Methanol	Seeds	All methods were found to be significant with FRAP exhibiting the highest scavenging activity at 2.1 mg GAE/g	[60]
12	DPPH, ORAC	Methanol	Leaves	Both DPPH and ORAC experiments found that the methanolic extract of the leaves had a significant radical scavenging capacity of 937 16 and 2247 63 *μ*mol trolox equivalent/g.	[38]

Notes: ALT = alanine aminotransferase, AST = aspartate transaminase, S/N = serial number, *D. microcarpum* = *Detarium microcarpum*, FRAP = ferric reducing antioxidant power, TBIL = total bilirubin, DPPH = 2,2-diphenyl-1-picrylhydrazyl, 2,20-azino-bis-3-ethyl-ethylbenzothiazoline-6-sulphonate, *SRASA* = superoxide radical anion scavenging assay, DDA = deoxyribose degradation and LPM = lipid peroxidation models, ABTS = radical cation scavenging assay, ORAC = oxygen radical absorbance capacity, IC_50_ = the half maximal inhibitory concentration, and TBIL = total bilirubin test.

**Table 3:** Anti-inflammatory activity of *D. microcarpum*.

S/N	Method	Solvents	Part of the plant	Major findings	Reference
1	*In Vivo*	n-hexane, ethylacetate, methanol, and aqueous	Leaf	Methanolic leaves extract showed the highest acute inflammatory activity at *p* < 0.05	[61]
2	*In Vivo*	Methanol	Stem bark	When injected intraperitoneally in mice, the LD_50_ of the extract was 471.2 mg/kg body weight and ≥5000 mg/kg body weight. The methanolic extract reduced the mean diameter of the rat paw in carrageenan-induced inflammation when compared to the normal saline-treated group.	[62]
3	*Egg albumin, formalin induced inflammation*	Methanol	Leaf	The results indicate that the methanol leaf extract exhibits considerable dose-dependent anti-inflammatory action against egg albumin and formalin-induced inflammation at *p* < 0.05. At doses of 200 and 400 mg/kg, the extract inhibited egg albumin by 26.5 and 29.4%, respectively, at the 5th hour. For all doses 200 and 400 mg/kg, the % inhibition of formalin-induced edema was 32.5.	[63]
4	*IN Vivo*	n-Hexane, ethylacetate, methanol, and aqueous	Leaf	Methanolic leaves extract showed the highest inhibition analgesic activity at 88%	[61]
5	*In Vivo*	Aqueous	Stem bark	Plasma activity was significantly lower in the groups treated with the extract at *p* < 0.05. In the same group, a low plasma level of total protein was also detected. As a result, oral administration of the aqueous bark extract demonstrated antiatherogenic, cardioprotective, and hepatoprotective activity.	[64]

Notes: S/N = serial number and LD_50_ = lethal dose.

**Table 4:** Antibacterial activity of *D. microcarpum*.

S/N	Method	Solvents	Part of the plant	Major findings	Reference
1	Agar-disc	Ethanol	Leaves	*S. aureus* and *S. salmonellae* were found to be resistant to all portions of the leaf extract tested at 15, 30, and 60 *μ*g/mL, respectively.	[49]
2	Agar well diffusion	Ethanol	Stem bark	At various doses of 100, 50, 25, and 12 mg/mL, respectively, the stem bark ethanolic extracts showed antibacterial activity against the studied species, with *S. aureus* having the largest zone of inhibition at 21 mm at 100 mg/mL.	[44]
3	*Agar disc diffusion method*	Methanol	Stem bark	The stem bark extract were highly active against the test strains, exhibiting substantial efficacy at 25 *μ*g/mL.	[65]
4	Microdilution	Methanol	Fruits	*Salmonella typhi, Salmonella enteritidis*, and *Salmonella typhimurium* were all inhibited by the microcarposide with the inhibition zone of 153.4, 76.7, and 76.7 *μ*M, respectively.	[45]
5	Broth dilution, *In vivo*	Ethanol	Root bark	Ethanolic root bark extract and the isolated compound rhinocerotinoic acid showed good efficacy *in vitro* and infected animals at an effective dose of 75 mg/kg.	[66]
6	Agar and disc diffusion	Petroleum ether	Seeds	Seeds were found to be significant against all eight tested strains with the highest zone of inhibition against *S. aureus* at 8.8 mm	[26]
7	Ager plate	Petroleum ether, chloroform, and ethanol	Bark	The ethanolic bark extract exhibited the highest activity against *Listeria monocytogenes* at 13 mm	[33]
8	MIC		Stem bark	Catechin gallate and methyl gallate compound 2 (MIC 200 *μ*g/mL) showed anti-MRSA activity, which is interesting because compounds 1 and 2 also showed anti-MRSA activity.	[40]
9	Agar disk diffusion and broth micro dilution		Essential oil	The extract showed moderate and strong inhibition zones of 12 and 22 mm against all of the tested microbial strains, respectively.	[28]
10	Ager well	n-hexane, ethyl acetate, chloroform, and methanol	Stem bark	Stem bark extract exhibited strong activity against *Staphylococcus* against the zone of inhibition 28 mm	[67]
11	MIC	Dichloromethane and methanol		The dicholoromethane extract of *Detarium microcarpum is* the most effective in inhibiting the growth of *Pythium aphanidermatum* at 75%.	[68]
12	*Disc diffusion*	Ethanol	Stem barks and seeds	The greatest inhibitory concentration was 100 mg/mL in proteus mirabilis, with an 8-mm inhibition zone.	[69]
13	*Mic*	Petroleum ether	Seed	The extract exhibited the growth of all tested bacteria significantly	[13]
14		Aqueous and methanol	Seeds	Highest zone of inhibition was recorded *against E. coli* at 18 mm	[11]

Notes: S/N = serial number and MIC = minimum inhibitory concentrations.

**Table 5:** Antifungal activity of *D. microcarpum*.

S/N	Method	Solvents	Part of the plant	Major findings	Reference
1		Petroleum ether	Seed	The extract inhibited the growth of all tested fungal significantly	[13]
2			Fruits pulp	Inhibited the growth of the tested fungal strain at 100 *μ*g	[24]
3		Hexane, methanol	Stem bark	The methanolic stem bark exhibited the highest inhibition against *Microsporum canis* at 10.70 mm.	[70]
4		Aqueous	Stem bark	The extract protects against castor oil-induced diarrhea by 53%, compared to the standard 91%.	[71]
5		Aqueous	Stem bark	The aqueous extract alone lowered the contractile amplitude of jejunal tissue dosage independently. Additionally, the aqueous extract lowered the contractile amplitude of an isolated jejunal segment subjected to 0.2 ml of acetylcholine at a concentration of 10 *μ*g/mL.	[72]

Notes: S/N = serial number.

**Table 6:** Antiparasitic activity of *D. microcarpum*.

S/N	Method	Solvents	Part of the plant	Major findings	Reference
1	Antiparasitic	Methanol	Stem bark	For the 200, 400, and 800 mg/kg tested doses, the methanolic stem bark extract had a significant (*p* < 0.001) curative as well as prophylactic impact, and the survival period of mice was significantly (*p* < 0.001) extended in the treated groups. There was no effect of the extract on the biochemical and haematological tests at *p* > 0.05.	[42]
2		Methanol	Leaf	When compared to the negative control, the methanolic leaf extract significantly reduced the average % of parasitemia level in the treatment group. At doses of 250, 500, and 1000 mg/kg, the extract cleared parasites at 83.52, 86.65, and 87.21%, respectively.	[73]
3		Methanol	Leaves	After 14 days of treatment, both doses of the extract administered significantly reduced parasitemia. On the other hand, neither dose of the extract reversed trypanosome-induced anaemia. Similarly, the extract was unable to improve hepatomegaly and splenomegaly caused by trypanosomes.	[74]
4		Methanol	Leaves	The observed result indicated that the methanolic leaf extract possesses trypano suppressive action, implying that it may be employed as a candidate for the development of medications to treat disorders caused by trypanosomes.	[75]
5		Methanol	Leaf	The methanolic leaf extract's of oral LD_50_ was determined to be greater than 5000 mg/kg. At all doses examined, the extract had substantial curative, suppressive, and preventive effects at *p* > 0.001. Additionally, the extract increased the survival period of treated mice by up to 19 days when compared to the negative control group.	[76]
6		Methanol and aqueous	Stem bark	Both the aqueous and methanolic stem bark extracts inhibited larval mobility in *C. elegans*, *Bristol, and C. elegans* DA1316 with a significant difference at *p* < 0.05 compared to the negative control.	[77]
	*Huh-7 Replicon assay*	Methanol	Stem bark	At a concentration of 10 *μ*M, the extract displayed strong inhibitory potency of 83.87 against hepatitis C virus, compared to control (RS446 (2-Me-C) at 86.76% respectively.	[78]

Notes: S/N = serial number.

**Table 7:** Antidiabetic activity of *D. microcarpum*.

S/N	Method	Solvents	Part of the plant	Major findings	Reference
1		Aqueous	dAgNps (leaves)	It was determined that dAgNps suppressed PANC-1 in the cell viability assay, with IC_50_ values of 84 *μ*g/mL	[79]
2	*In Vivo*	Aqueous	Seeds	There was no substantial health risk associated with the administration of 200 mg·kg^−1^ of the methanolic seeds extract, alone or in combination with *B. eurycoma* and *M. pruriens* seeds, in the current investigation.	[80]
3	*In Vivo*		Root	The root extract considerably lowered the blood sugar levels of alloxan diabetic rats at (*p* < 0.05).	[15]
4	*In Vivo*	Methanol	Leaf	At 500, 750, and 1000 mg/kg body weight, the methanolic leaf extract significantly reduced blood glucose levels. Compared to diabetic rats administered with the conventional medication (glibenclimide), the extract significantly increased the body weight at (*p* < 0.05)	[81]
5	*In Vivo*	n-Hexane, ethyl acetate, and 70% methanol	Stem bark	The extracts were found to have anti-implantation activity, which supports its usage as a contraceptive in traditional medicine.	[82]
6	*In Vitro*	70% methanol	Leaf	On-amylase and -glucosidase IC_50_ of leaf extracts at 63 and 158 *μ*g/mL, respectively, were lower than those of acarbose.	[57]
7	*In Vivo*	Aqueous	Seeds	The seeds' extract lowers postprandial blood glucose and insulin levels in humans.	[83]
8	*In Vivo*	Aqueous	Seeds	Significant reductions in blood sugar and cholesterol levels were observed with the seed extract. The extract exhibited no influence on haematological or blood chemistry indicators, demonstrating its safety.	[84]
9	*In Vivo*	Methanol	Leaf	When compared to glibenclamide, methanol leaf extract (200 and 400 mg/kg body weight) caused a significant dose-dependent drop in blood sugar levels, 20.62 and 40.75%, respectively, and a considerable recovery of body weight in diabetic rats after four hours of administration (57.49%) was observed.	[85]
10	*In Vivo*		Gum	The results indicated that detarium gum is a superior excipient for the formulation of metformin mucoadhesive delivery systems. Additionally, gum has shown a promising antidiabetic impact and should be taken with caution, since it may result in abnormally low blood glucose levels.	[86]
11	*In Vivo*	Methanol	Seed	The methanolic seed extract lowered alpha-amylase and glucosidase at 69.3 and 31.1 mg/mL, respectively	[60]
12	*In Vivo*		Bread	Reduced incremental blood glucose and postprandial glucose levels by a statistically significant amount at *p* < 0.05. Glucose has a 62% area-under-curve.	[87]

Notes: S/N = serial number.

**Table 8:** Insecticidal potential of *D. microcarpum*.

S/N	Method	Solvents	Part of the plant	Major findings	Reference
1	Wheat wafer disc bioassay	n-Hexane, and methanol	Leaves, stem, and root barks	With an LC_50_ value of 47 *μ*g/insect, methanol extract of the root bark had the strongest antifeedant index and contact toxicity effect on *T. casteneum,* hbst.	[35]
2	Paper disk	Methanol	Leaves	3,13E-clerodien-15-oic acid, 4 (18), 13E-clerodien-15-oic acid, 18-oxo-3,13E-clerodien-15-oic acid, and 2-oxo-3,13E-clerodien-15-oic acid are the active chemicals. Except for the latter, this is the first report of these compounds from D. microcarpum. The four compounds had considerable antifeedant activity at 1%.	[41]

Notes: S/N = serial number.

**Table 9:** Anticancer activity of *D. microcarpum*.

S/N	Method	Solvents	Part of the plant	Major findings	Reference
9	Presto blue cell viability assay	Methanol and aqueous	Stem bark	The IC_50_ values for the methanol and aqueous extracts of the three plants inhibiting MCF7 cell growth ranged from 78 to >500 *μ*g/mL. The strongest antioxidant and antiproliferative activity was observed in stem bark extracts. As a result, the stem bark of these plants may contain anti-breast cancer chemicals.	[59]
		Aqueous	Leaf and stem	The leaf extracts inhibited the cancer cell, the percentage inhibition increased as well, reaching 89.47% optimal dosage of 100 mg/cm^3^. However, this research has revealed leaf's promise as a cancer therapeutic, particularly in developing nations, and has brought new knowledge to science.	[88]
		Aqueous	dAgNps (leaves)	HeLa cell growth was decreased by dAgNps with IC_50_ values of 31.5 *μ*g/mL.	[79]

Notes: S/N = serial number.

**Table 10:** Toxicity evaluation of *D. microcarpum*.

S/N	Method	Solvents	Part of the plant	Major findings	Reference
1	*In Vivo*	Methanol	Leaf	Toxicological tests have established that a dose of 5000 mg/kg of methanolic leaf extract is safe.	[63]
2	*In Vivo*	70% methanol	Stem bark	According to the study, prolonged use of the extract in the management of medical conditions may have a harmful effect on certain essential organs.	[89]
3	*In Vivo*	Aqueous	Stem bark	It may be concluded that supplementation with aqueous stem bark extract was useful in moderating the changes in liver, kidney, and serum variables of rats exposed to mycotoxins.	[90]
4	*In Vivo*	Methanol	Stem bark	The methanolic stem bark extract, which reveals that prolonged use of the extract in the therapy of disease conditions may be related to some unfavourable effects on some essential organs.	[12]
5	*In Vivo*	Aqueous	Fruit	Fruit flour was nutritious as a food additive up to 30% without causing clinical indications or death.	[88]
6	*In Vivo*	n-butanol	Stem bark	All treatment groups had a substantial decrease in relative liver weight at *p* < 0.05 when compared to the negative control. These findings imply that *Detarium microcarpum* stem bark contains antioxidant phytochemicals and may be useful in the treatment of liver damage.	[91]
7	*In Vivo*		Fruits	The results of this experiment indicate that the fruit of *D. microcarpum* may have an adverse effect on rats and may include antinutrients that harm its digestion and absorption, resulting in the observed retardation of growth in rats.	[92]
8	*In Vivo*		Seeds	The inclusion of the seeds in the diet, had no effect on the haematological and biochemical indicators at *p* > 0.05. Thus, tallow seed meal can be used in broiler chicken feeds up to 20% without affecting organ weight, haematological, or biochemical indices of broiler chickens.	[93]
9	*In Vivo*		Fruits	Increased incorporation of seeds, reduced weight gain and FCR in a linear fashion. Birds fed 10%, 15%, and 20% diets had lower haematological and serum biochemical indices than those fed 5% and control diets at *p* < 0.05. Broiler chicks' blood components were not adversely affected by the inclusion of 5% DSM in their diets. Processed DSM must be added to broiler meals to increase their incorporation levels above 5%.	[94]
10	BST	Methanol	Stem bark	It was shown that the LC_50_ for brine shrimp larvae was 158.49 g/mL for the methanolic extract of the stem bark. The results show that *Detarium microcarpum* is toxic and thus not safe, especially when administered in large doses without proper monitoring and management.	[95]
11	MTT	Hexane, chloroform, ethyl acetate, and methanol	Fruits pulp	The chloroform and ethyl acetate extracts of *D. microcarpum* fruit pulp were the most cytotoxic to normal fibroblasts in a biocompatibility investigation of the fruit pulp. More than 80 percent of cells died when the hexane extract was applied at 500 g/mL, which was the highest concentration that was found to be cytotoxic. The cytotoxic effects of methanol extract were negligible.	[96]
12	MTT	Ethanolic	Fruit pulp	Human lymphocytes were not harmed by the fruit pulp ethanol extract. The cytotoxicity of hydrogen peroxide and tert-butyl hydroperoxide to human lymphocytes was also dramatically lowered by pretreatment with fruit extracts. In terms of cytoprotective efficacy, both the extract and ascorbic acid were comparable at *p* > 0.05.	[55]
13	*In Vivo*	Methanol	Stem bark	Mice died at concentrations of 2900 mg/Kg and 1600 mg/kg body weight from the methanolic stem bark extract, with an LD_50_ value of 3,807.89 mg/kg. Experiments with extracts of these plants have yielded a good dose guidance for an ongoing antimalarial study.	[43]
14	Brine shrimp lethality test	Dichloromethane and methanol		The methanol extract of *Detarium microcarpum,* had an LC_50_ of 1540.00 *μ*g/mL (non-toxic).	[68]
15	*In Vivo*	Methanolic	Leaf	The methanolic leaf extract showed that none of the organs showed any histological changes during the monitoring period.	[81]
16	*In Vivo*	Ethanol	Fruits	The fruit's antioxidant molecules scavenging the hydroxyl radical, as well as the peroxyl and alkoxyl radicals produced by lipid peroxidation had a gene protective impact.	[53]
17	*In Vivo*	Methanolic	Leaf	The oral LD_50_ of the extract was determined to be greater than 5000 mg/kg of body weight. There were no significant differences between the therapy groups when it came to renal function and haematological analysis.	[73]

Notes: S/N = serial number.

## Data Availability

The data supporting the current study are given in the article.

## Abbreviations

ALT: Alanine aminotransferase
AST: Aspartate transaminase
dAgNps: Silver nanoparticles
*D. microcarpum*n: *Detarium microcarpum*
FRAP: Ferric reducing antioxidant power
TBIL: Total bilirubin
DPPH: 2,2-diphenyl-1-picrylhydrazyl, 2,20-azino bis-3-ethyl-ethylbenzothiazoline-6-sulphonate
SRASA: Superoxide radical anion scavenging assay
DDA: Deoxyribose degradation
LPM: Lipid peroxidation models
ABTS: Radical cation scavenging assay
MIC: Minimum inhibitory concentrations
MBC: Minimum bacterial concentration
HPLC: High-performance liquid chromatography
HPTLC: High-performance thin-layer chromatography
GCMS: Gas chromatography mass spectrometry
FTIR: Fourier transform infrared.
